# Calcium and Calmodulin Are Involved in Nitric Oxide-Induced Adventitious Rooting of Cucumber under Simulated Osmotic Stress

**DOI:** 10.3389/fpls.2017.01684

**Published:** 2017-09-27

**Authors:** Lijuan Niu, Jian Yu, Weibiao Liao, Jihua Yu, Meiling Zhang, Mohammed M. Dawuda

**Affiliations:** ^1^College of Horticulture, Gansu Agricultural University, Lanzhou, China; ^2^College of Science, Gansu Agricultural University, Lanzhou, China; ^3^Department of Horticulture, Faculty of Agriculture, University for Development Studies, Tamale, Ghana

**Keywords:** abiotic stress, adventitious rooting, nitric oxide, calcium, chlorophyll fluorescence, antioxidant system

## Abstract

Osmotic stress is a major form of abiotic stress that adversely affects growth and development of plants and subsequently reduces yield and quality of crops. In this study, the effect of nitric oxide (NO) and calcium (Ca^2+^) on the process of adventitious rooting in cucumber (*Cucumis sativus* L.) under simulated osmotic stress was investigated. The results revealed that the effect of exogenous NO and Ca^2+^ in promoting the development of adventitious roots in cucumber seedlings under simulated osmotic stress was dose-dependent, with a maximal biological response at 10 μM NO donor nitroprusside (SNP) or 200 μM Ca^2+^. The application of Ca^2+^ chelators or channel inhibitors and calmodulin (CaM) antagonists significantly reversed NO-induced adventitious rooting, implying that endogenous Ca^2+^/CaM might be involved in NO-induced adventitious rooting under osmotic stress. Moreover, intracellular Ca amount was also increased by NO in cucumber hypocotyls during the development of adventitious roots under osmotic stress. This increase of endogenous Ca^2+^ was inhibited by NO specific scavenger 2-(4-carboxyphenyl) -4,4,5,5-tetramethylimidazoline-1-oxyl-3-oxide potassium salt (cPTIO), nitrate reductase inhibitors tungstate (Na_2_WO_4_) and sodium azide (NaN_3_)_._ This gives an indication that Ca^2+^ might be a downstream signaling molecule in the adventitious root development by NO under osmotic condition. The results also show that NO or Ca^2+^ play a positive role in improving plant water status and photosynthetic system by increasing chlorophyll content and photochemical activity in leaves. Furthermore, NO and Ca^2+^ treatment might alleviate the negative effects of osmotic stress by decreasing membrane damage and reactive oxygen species (ROS) production by enhancing the activities of superoxide dismutase (SOD), catalase (CAT) and ascorbate peroxidase (APX). Therefore, Ca^2+^/CaM may act as a downstream signaling molecule in NO-induced development of adventitious root under simulated osmotic stress through improving the photosynthetic performance of leaves and activating antioxidative system in plants.

## Introduction

Nitric oxide (NO), as a free radical gas, has been synthesized enzymatically or non-enzymatically (Skiba et al., 1993; Rockel et al., 2002). Previous reports have indicated that NO might regulate the growth and physiological processes in plants, including seed germination (Beligni and Lamattina, 2000; Wang et al., 2015), root growth and development (Liao et al., 2011; Zhao et al., 2015), senescence (Liao et al., 2013), stomatal closure (Neill et al., 2002; Shi C. et al., 2015) and the growth of pollen tube (Wang et al., 2009). It has also been reported that NO plays an essential role in response to various abiotic stresses (Garcia-Mata and Lamattina, 2001; Salgado et al., 2013; Kaur et al., 2015; Liu W. et al., 2015). Besides, an increasing body of evidence indicated that NO served as a regulator in plant response to osmotic stress. Liao et al. (2012a) found that exogenous NO improved the development of adventitious roots in marigold explants under drought stress conditions by improving photosynthesis and carbohydrate and nitrogen contents. Shan et al. (2015) have reported that jasmonic acid (JA) alleviated drought stress by inducing endogenous NO, which could up-regulate the activity of ASA-GSH cycle. In addition, Jday et al. (2016) found that exogenous application of NO increased drought tolerance and mitigated damage by regulating proline metabolism and reducing oxidative damage by increasing the activities of superoxide dismutase (SOD) and catalase (CAT) in *Cakile maritima*.

Increasing evidence pointed out that the change of intracellular Ca^2+^ concentration is a mark of signaling transduction to mediate various cellular processes in plants (Kong et al., 2015; Tang et al., 2015). Recently, it has been reported that Ca^2+^ regulates the processes of growth and development in plants including seed germination (Kong et al., 2015), pollen tube growth (Zhou et al., 2014), and root growth (Liao et al., 2012b). Some studies have indicated that variations in cytosolic free Ca^2+^ concentration might be involved in plant response to different kinds of abiotic stresses (Zou et al., 2015; Li et al., 2016). Ca^2+^ plays a role in mediating plant adaptation to drought stress condition. For instance, Zou et al. (2010) found that Ca^2+^ could mediate stomatal movements in *Arabidopsis* plants under drought stress through calcium-dependent protein kinases (CDPKs). Application of Ca^2+^ also reduced drought-induced proline accumulation, which implied that Ca^2+^ played a role in response to drought stress in *Triticum aestivum* L. (Sadiqov et al., 2002). However, the mechanism of Ca^2+^ signaling in regulating plant growth and response to abiotic stress still needs further investigation.

The interaction of NO and Ca^2+^ has been regarded as a critical regulator in plant growth and development and in response to abiotic stress. For example, Lanteri et al. (2006) reported that Ca^2+^ is involved in NO-induced adventitious root formation in cucumber. Chen and Kao (2012) found that Ca^2+^ was involved in NO-induced formation of lateral roots (LR) in rice. Excluding endogenous Ca^2+^ inhibited the NO-induced LR formation. However, the authors did not find any relationship between Ca^2+^ and endogenous NO during LR formation. A crosstalk between NO and Ca^2+^ in inducing adventitious rooting in marigold under normal condition has been reported (Liao et al., 2012b). In addition, Ca^2+^ signaling induced endogenous NO accumulation by inducing hydrogen peroxide (H_2_O_2_) generation during stomatal closure in *Arabidopsis* guard cells (Wang et al., 2012). Xu et al. (2016) also found an interaction between NO and Ca^2+^ under high irradiance in tall fescue leaves. The occurrence of a crosstalk between NO and Ca^2+^ under copper stress was also found in *Ulva compressa* (González et al., 2012). NO generation under copper stress might be dependent on Ca^2+^ release through various Ca^2+^ channels, which were also activated by NO (González et al., 2012). Cellular responses to NO and Ca^2+^ signaling are complicated, therefore, further research to deepen our understanding of the crosstalk between NO and Ca^2+^ in plants is needed.

Osmotic stress as a situation which might prevent plants from absorbing enough water induces the inhibition of plant growth and oxidative damage (Jiang et al., 1993). The common osmotic stresses include drought, salt and cold stresses. It has been reported that osmotic stress significantly reduced the fresh weight and water content in leaf blade and leaf petiole of sugar beet (*Beta vulgaris* L.) (Wu et al., 2016). Osmotic stress interfered with various metabolic processes (Búfalo et al., 2016) in plants such as photosynthesis (Bündig et al., 2016) and respiration (Zorrilla-Fontanesi et al., 2016). Previous study has shown that Ca^2+^ and CDPK could be involved in adventitious rooting, which was induced by NO in cucumber (Lanteri et al., 2006). However, Liao et al. (2012b) indicated that NO induced adventitious root development in marigold through enhancing endogenous Ca^2+^ and CaM level under stress-free conditions. The role of NO and Ca^2+^ in adventitious rooting under abiotic stress is unknown. We conduct this experiment with the hypothesis that NO, Ca^2+^ and their crosstalk may affect adventitious development in plants under abiotic stress. The objective of this study was to elucidate the potential role of NO and Ca^2+^ in adventitious rooting process under osmotic stress condition. In this study, we provide evidence that Ca^2+^/CaM are required for NO-induced adventitious root development in cucumber under osmotic stress and this improves our understanding of the mechanism of NO signaling transduction under abiotic stress.

## Materials and Methods

### Plant Materials

Cucumber (*Cucumis sativus* ‘Xinchun 4’) seeds were germinated in petri dishes on filter papers moistened with distilled water and maintained at 25 ± 1 C for 6 days with a 14 h photoperiod (photosynthetically active radiation = 200 μmol s^-1^ m^-2^). Primary roots of 6 days old seedlings were removed and the cucumber explants were then maintained under the same conditions of temperature and photoperiod for another 6 days in the presence of different media as indicated below. These media were changed every day in order to keep the solution fresh. Root number and length per explant were counted and measured.

### Treatments of Explants

Experiment 1: Polyethylene glycol 6000 (PEG 6000, Shanghai Chemical Reagent Co. Ltd., Shanghai, China) was used to simulate osmotic stress. Explants were placed in Petri dishes containing filter paper moistened with distilled water (control) and different concentrations of PEG 6000, sodium nitroprusside (SNP, a donor of NO, Merck, Darmstadt, Germany) and calcium chloride (CaCl_2_, Solarbio, Beijing, China) and kept at 25 ± 1°C. The following chemicals were added with suitable concentration of PEG, SNP, or CaCl_2_:50 μM *S*-nitroso-*N*-acetylpenicillamine (SNAP, a donor of NO, Sigma, United States), 50 μM K_4_Fe(CN)_6_ (SNP analog, Solarbio, Beijing, China), 100 μM sodium nitrate (NaNO_3_, degradation product of SNP, Solarbio, Beijing, China).

Experiment 2: Ca chelators and channel inhibitors and CaM antagonists: (1) 100 μM ethylene glycol-*bis* (2-aminoethylether)- *N,N,N′,N′*-tetraacetic acid (EGTA, Sigma, United States): a Ca^2+^ chelator (Liao et al., 2012b); (2) 30 μM 1,2-*bis* (o-aminophenoxy) ethane-*N,N,N′,N*′-tetraacetic acid tetra (acetoxymethyl) ester (BAPTA/AM, Santa Cruz, CA, United States; BAPTA-AM was prepared for 15 min at -20°C before the experiment): a membrane permeable Ca^2+^ chelator (Liao et al., 2012b); (3) 80 μM *N*-(6-aminohexyl)-5-chloro-1-naphthalenesulfonamide hydrochloride (W-7, Santa Cruz, CA, United States): a CaM antagonist (González et al., 2012); (4) 80 μM *N*-(6-aminohexyl)-1-naphthalenesulfonamide (W-5, Santa Cruz, CA, United States): a CaM antagonist (Liao et al., 2012b); (5) 100 μM trifluoperazine dihydrochloride (TFP, Santa Cruz, CA, United States): a CaM antagonist (Lanteri et al., 2006); (6) 500 μM lanthanum chloride (LaCl_3_, Solarbio, Beijing, China): a Ca^2+^ channel blocker (Lanteri et al., 2006); (7) 150 μM nifedipine (Solarbio, Beijing, China): a Ca^2+^ channel blocker (Reiss and Herth, 1985).

Experiment 3: NO scavengers and nitrate reductase inhibitors: (1) 200 μM 2-(4-carboxy-2-phenyl)-4, 4, 5, 5-tetramethylimidazoline-1-oxyl-3-oxide (cPTIO, Sigma, United States): a NO specific scavenger (Liao et al., 2012b); (2) 10 μM sodium azide (NaN_3_, Solarbio, Beijing, China): a nitrate reductase inhibitor (Liao et al., 2013); (3) 100 μM sodium tungstate (Na_2_WO_4_, Solarbio, Beijing, China): a nitrate reductase inhibitor (Tian et al., 2007). The concentrations of these chemicals were based on the results of a preliminary experiment.

### Cytosolic Free Ca^2+^ Observation

Fluo-3/AM (Sigma, United States) as molecular probes was utilized to determine intracellular Ca level in plant. 20 μM Fluo-3/AM was loaded into cucumber hypocotyls (about 100 – 200 mm long) at 4°C in the dark (Zhang et al., 1998). After 2 h of incubation, the hypocotyls were washed three times with distilled water to remove the excess of fluorescence and place under 20°C for 1 h. The Fluo-3 fluorescence of the hypocotyls after 48 h of treatments was visualized via fluorescence microscope (Leica 400 ×, Planapo, Wetzlar, Germany), for an excitation wavelength of 488 nm and an emission wavelength of 520–530 nm. After that, the fluorescence intensity of endogenous Ca^2+^ at 48 h was measured via Image Pro software (Media Cyberntics, United States).

### Determination of Endogenous NO Production

Fluorescent probe 4-amino-5-methylamino-2′,7′-diamino-fluoresceindiacetate (DAF-FM DA) was utilized to measure the level of endogenous NO in cucumber hypocotyls after 48 h of treatments. The hypocotyls were loaded with 10 μM DAF-FM DA (Graziano and Lamattina, 2007) in 50 mM Tris-HCl (pH 7.4) for 2 h in the dark. Then, the samples were washed three times with fresh buffer for 15 min. DAF-FM DA fluorescence was visualized via fluorescence microscope (Leica 400 ×, Planapo, Wetzlar, Germany), for excitation with the 488 nm, and emission with 500 – 530 nm.

### Measurement of Water Potential

The water potential of cucumber leaves was directly determined using a dew-point water potential meter WP4T (Decagon Devices, United States). The measurements of water potential in cucumber leaves after 48 h of treatment were made at 9:00 – 10:00 am.

### Determination of TBARS, H_2_O_2_ and O_2_^-^ Content

TBARS content was determined as described by Cakmak and Horst (1991). 0.2 g fresh explant was grounded with 3 mL of 0.1% (w/v) trichloroacetic acid (TCA). The homogenate was centrifuged at 12000 *g* for 5 min, 1 mL supernatant was added to 4 mL of 20% (w/v) TCA which included 0.5% (w/v) TBA. Then, samples were incubated in a water-bath (30 min) at 90°C. After that, the reaction was incubated in ice bath. After 10000 *g* centrifugation for 5 min, the absorbance of the supernatant was recorded at 532 and 600 nm.

H_2_O_2_ content was determined the method of Velikova et al. (2000). 0.2 g sample was homogenized with 4 mL of 0.1% (w/v) trichloacetic acid in an ice bath. After 12000 *g* centrifugation at 4°C for 15 min, the absorbance of the mixture reaction which includes 0.5 mL of the supernatant, 0.5 mL of 10 mM potassium phosphate buffer (pH 7.0) and 1 mL of 1 M potassium iodide was recorded at 390 nm.

The rate of superoxide production (O_2_^-^) was measured according to Chaitanya and Naithani (1994). Fresh cucumber explant (0.2 g) was homogenized with 100 mM sodium–phosphate buffer (pH 7.8) in an ice bath. The centrifugation was performed for 20 min at 4°C. The mixture includes 0.5 mL of 100 mM sodium phosphate buffer (pH 7.8), 0.5 mL of 1 mM hydroxylammonium chloride and 0.5 mL of supernatant. Then, 1 mL of 17 mM sulfanilic acid and 1 mL of 7 mM α-naphthylamine were added in the reaction which was maintained at 25°C for 20 min. The absorbance of sample was measured at 540 nm. ROS production was measured after 48 h of treatments.

### Determination of Chlorophyll Content

About 0.2 g leaves were ground to a fine powder and extracted with 5 mL of 80% acetone (v/v). The amounts of Chlorophyll a, b, or a + b after 48 h of treatments were determined by spectrophotometer, measuring the absorbance at 645 and 663 nm and the chlorophyll concentration (mg/g DW) was calculated by using the equations according to Arnon (1949).

### Measurement of Chlorophyll Fluorescence

This was measured as described by Genty et al. (1989). Fluorescence parameters of cucumber leaves after 48 h were measured using chlorophyll fluorescence imaging system (MAXI Imaging-PAM, Walz, Effeltrich, Germany) at 25°C. The intensities of the modulated measuring beam, actinic light and saturating light were 0.1 μmol m^-2^ s^-1^, 81 μmol m^-2^ s^-1^ and 2700 μmol m^-2^ s^-1^ PFD, respectively. The duration of saturation pulses was 0.8 s. Meanwhile, the duration of actinic light was set to 5 min to obtain chlorophyll fluorescence under the steady state condition. After putting the plant samples to 30 min dark period adaptation, the initial fluorescence yield (*F_O_*) was obtained. The maximum fluorescence yield (*Fm*) was measured with the application of a saturation pulse. Variable chlorophyll fluorescence (*Fv*) was evaluated as *Fv* = *Fm* – *Fo*. The maximum quantum yield of PSII (*Fv* / *Fm*) were calculated as *Fv* / *Fm* = (*Fm* – *Fo*) / *Fm*. Maximum fluorescence yield of the light adapted leaf (*Fm’*) were obtained after the application of a saturation pulse and *Fs* represented steady-state chlorophyll fluorescence. Effective quantum yield of PSII (ΦPSII) = (*Fm′*- *Fs*)/*Fm′*. After turning off action light, far red light (10 μmol m^-2^ s^-1^) was illuminated immediately obtaining the minimum fluorescence yield in light-adapted state (*Fo′*). Photochemical quenching (qP = [*Fm′*- *Fs*]/[*Fm′* – *Fo′*]) and non-photochemical (NPQ = [*Fm* – *Fm′*]/*Fm′*]) quenching coefficients were calculated (Liu Z. et al., 2015).

### Antioxidant Enzyme Assays

Frozen explant (approximately 200 mg) were homogenized in 5 mL of 50 mM sodium phosphate buffer (pH 7.8) containing 5 mM ethylenediaminetetraacetic acid and 2% polyvinylpyrrolidone for SOD and CAT assay or the combination with the addition of 2 mM ascorbic acid (ASC) for ascorbate peroxidase (APX) assay. The homogenate was centrifuged at 12000 *g* for 20 min at 4°C and the supernatant was used as the crude enzyme extract. Total SOD activity was measured according to Dhindsa et al. (1981). The definition of one unit of SOD was the amount of crude enzyme extract required to inhibit the reduction rate of Nitrotetrazolium Blue chloride (NBT) by 50%. APX activity was evaluated by measuring the decrease in absorbance at 290 nm of a reaction mixture including 25 mM sodium phosphate buffer containing 0.1 mM ethylenediaminetetraacetic acid (pH 7.0), 20 mM H_2_O_2_, 5 mM ASC and 0.1 mL enzyme extract as previously described by Nakano and Asada (1981). According to Aebi (1984), CAT activity was evaluated by monitoring the consumption of H_2_O_2_ at 240 nm by a reaction mixture including 25 mM sodium phosphate buffer, 0.1 mM ethylenediaminetetraacetic acid (pH 7.0), 100 mM H_2_O_2_ and 0.1 mL enzyme extract. The activities of antioxidant enzymes were measured after 48 h of treatments.

### Statistical Analysis of the Data

All the results in the figures and table were expressed as the mean values ± SE from three independent replicates (10 samples per replication). Data was analyzed using the Statistical Package for Social Sciences for Windows (version 13.00; SPSS, Inc., Chicago, IC, United States). Analysis of Variance (ANOVA) was done and statistical differences among treatments were analyzed through Duncan’s multiple range test (*P* < 0.05).

## Results

### Effect of Exogenous SNP and CaCl_2_ on Adventitious Rooting under Osmotic Stress

**FIGURE 1:**
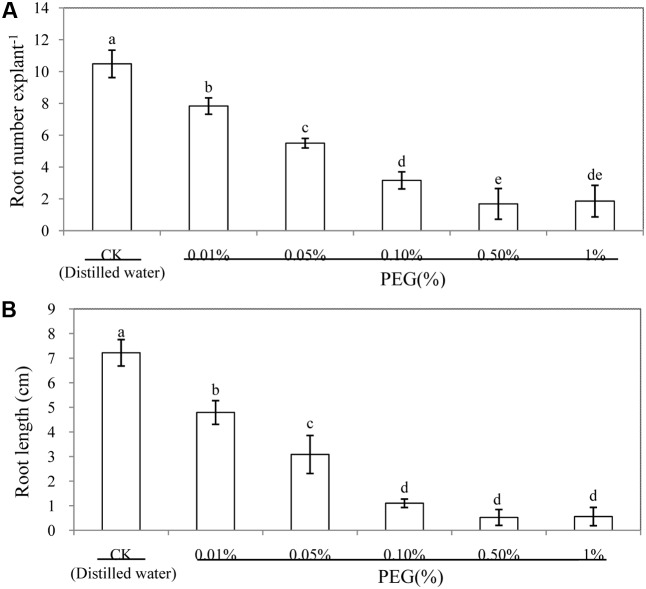
Effect of different concentrations of PEG on adventitious root development in cucumber explants. The primary roots were removed from hypocotyl of 6-day-old seedlings. Explants were incubated for 6 days with different concentrations of PEG 6000. The numbers **(A)** and root length **(B)** of adventitious root were expressed as mean ± SE (*n* = 3, 10 explants were used per replicate). Bars with different lower case letters were significantly different by Duncan’s multiple range test (*p* < 0.05).

To assess the effect of PEG on adventitious rooting in cucumber, we performed a dose-response experiment with PEG. When different concentrations of PEG solution were applied, the amount of adventitious roots and root length decreased significantly (**Figure 1**). As shown in **Figure 1**, root number and root length under 0.05% PEG decreased to about half that of the control treatment. Therefore, 0.05% PEG was utilized to simulate osmotic stress in the following experiments.

**FIGURE 2:**
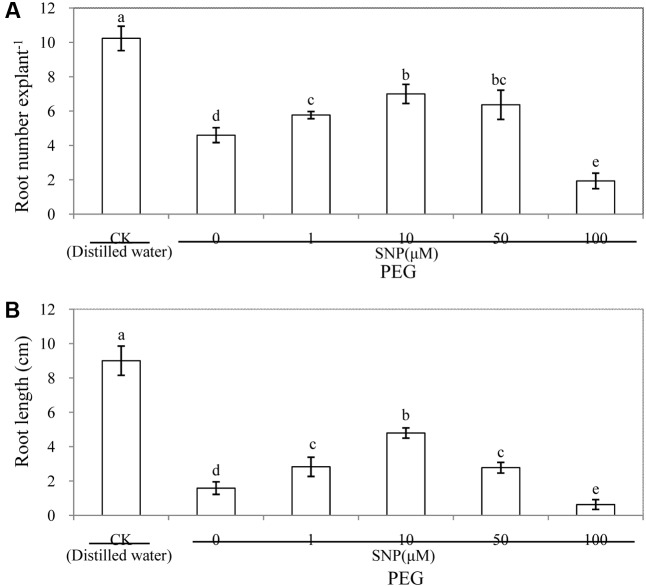
Effect of different concentrations of SNP on adventitious root development in cucumber explants under osmotic stress. The primary roots were removed from hypocotyl of 6-day-old seedlings. Explants were incubated for 6 days with distilled water (control) or 0.05% (w/v) PEG 6000 plus different concentrations of SNP. The numbers of adventitious root **(A)** and root length **(B)** were expressed as mean ± SE (*n* = 3, 10 explants were used per replicate). Bars with different lower case letters were significantly different by Duncan’s multiple range test (*p* < 0.05).

In order to investigate the effects of NO on the development of adventitious root under osmotic stress, cucumber explants were treated with different concentrations of SNP (a donor of NO). As shown in **Figure 2**, lower concentrations of SNP (1, 10, and 50 μM) treatments significantly increased the number of adventitious roots and root length under osmotic stress. However, a higher dose (100 μM SNP) significantly decreased the root number and root length, which indicates that the effect of NO on root number of adventitious roots was dose-dependent under osmotic stress, with a maximal biological response at 10 μM SNP (**Figure 2** and Supplementary Figure S1). Additionally, root numbers and root length of 10 μM SNP treatment increased by 52.2 and 201.9%, respectively, compared with those of PEG treatment. These results indicate that 10 μM SNP might significantly reverse the adverse effect of osmotic stress and promote the development of adventitious roots. Thus, 10 μM SNP was utilized for further experiments to study the processes of adventitious rooting under osmotic stress.

**FIGURE 3:**
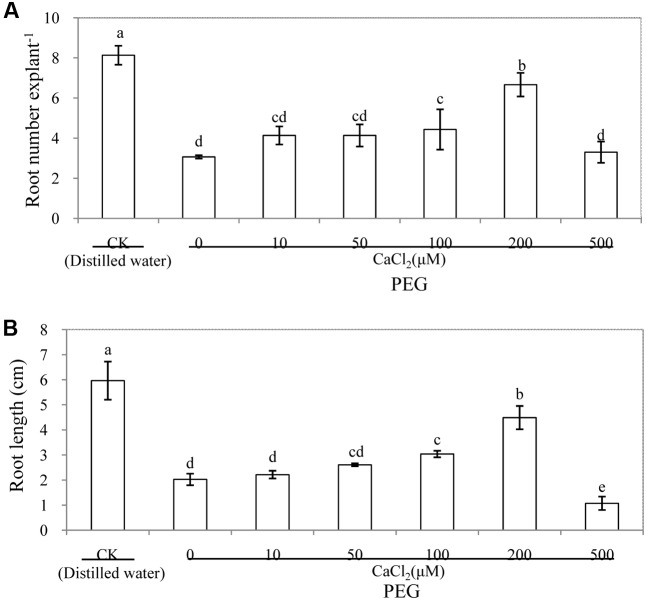
Effects of different concentrations of CaCl_2_ on adventitious root development in cucumber explants under osmotic stress. The primary roots were removed of 6-day-old seedlings. Explants were incubated for 6 days with distilled water (control) or 0.05% (w/v) PEG 6000 plus different concentrations of CaCl_2_. Adventitious root numbers **(A)** and root length **(B)** were expressed as mean ± SE (*n* = 3, 10 explants was used per replicate). Bars with different lower case letters were significantly different by Duncan’s multiple range test (*p* < 0.05).

As shown in **Figure 3**, the number and length of adventitious roots were significantly affected by different concentrations of CaCl_2_ treatments under osmotic stress. Exogenous CaCl_2_ treatments showed a concentration-dependent effect on adventitious rooting. There was no marked difference between 0, 10, and 50 μM CaCl_2_. Meanwhile, root number from 100 and 200 μM CaCl_2_-treated explants increased by 44.3 and 117.3%, respectively, compared with those of the PEG treatment. In addition, application of 200 μM CaCl_2_ significantly increased the root length by 121.2% in comparison with the PEG treatment alone. The number and length of adventitious roots decreased greatly at CaCl_2_ concentration of 500 μM (**Figure 3** and Supplementary Figure S2). Among the different concentrations of CaCl_2_ treatments, 200 μM CaCl_2_ significantly increased the root number and root length and had the greatest effect in promoting the development of adventitious root under osmotic stress. Therefore, we utilized 200 μM CaCl_2_ in the following experiments.

### Effect of Ca^2+^ Chelators, Channel Inhibitors and CaM Antagonists on Adventitious Rooting under Osmotic Stress

**FIGURE 4:**
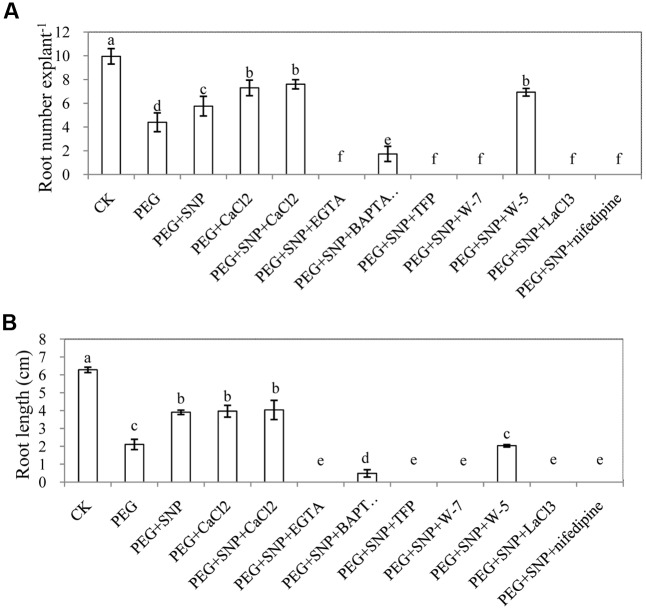
Effect of EGTA, BAPTA/AM, TFP, W-7, W-5, LaCl_3_ or nifedipine on adventitious rooting under osmotic stress. The primary roots were removed of 6-day-old seedlings. And then explants were incubated for 6 days with distilled water (control) or 0.05% (w/v) PEG 6000, PEG + 10 μM SNP, PEG + 200 μM CaCl_2_, PEG + 10 μM SNP + 200 μM CaCl_2_, PEG + 10 μM SNP + 100 μM EGTA, PEG + 10 μM SNP + 30 μM BAPTA/AM, PEG + 10 μM SNP + 100 μM TFP, PEG + 10 μM SNP + 80 μM W-7, PEG + 10 μM SNP + 80 μM W-5, PEG + 10 μM SNP + 500 μM LaCl_3_ or PEG + 10 μM SNP + 150 μM nifedipine. Adventitious root numbers **(A)** and root length **(B)** were expressed as mean ± SE (*n* = 3, 10 explants were used per replicate). Bars with different lower case letters were significantly different by Duncan’s multiple range test (*p* < 0.05).

In order to further investigate the requirement of Ca^2+^/CaM for NO-induced adventitious rooting, Ca^2+^ chelators (EGTA, BAPTA/AM), Ca^2+^ channel inhibitors (LaCl_3_ or nifedipine) and CaM antagonist (TFP, W-7 and W-5) were applied in this study (**Figure 4**). The results showed that EGTA, BAPTA/AM, LaCl_3_ and nifedipine significantly inhibited the development of adventitious roots under osmotic stress. Similarly, TFP and W-7 treatments also had a significant reduction in adventitious rooting. However, W-5, acting as a CaM antagonist, hardly reduced adventitious root number and length. SNP, CaCl_2_ and SNP + CaCl_2_ treatments obviously alleviated osmotic stress and promoted the development of adventitious roots (**Figure 4** and Supplementary Figure S3).

### Effect of K_4_Fe(CN)_6_, SNAP and cPTIO on Adventitious Rooting under Osmotic Stress

**FIGURE 5:**
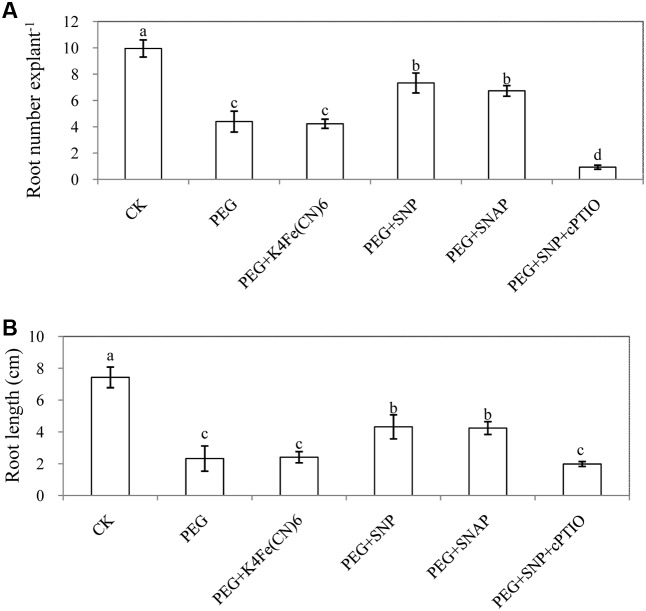
Effect of K_4_Fe(CN)_6_, SNAP or cPTIO on adventitious rooting under osmotic stress. The primary roots were removed of 6-day-old seedlings. And then explants were incubated for 6 days with distilled water (control) or 0.05% (w/v) PEG 6000, PEG + 50 μM K_4_Fe(CN)_6_, PEG + 10 μM SNP, PEG + 50 μM SANP, PEG + 10 μM SNP + 200 μM cPTIO. Adventitious root numbers **(A)** and root length **(B)** were expressed as mean ± SE (*n* = 3, 10 explants were used per replicate). Bars with different lower case letters were significantly different by Duncan’s multiple range test (*p* < 0.05).

Here, K_4_Fe(CN)_6_ and SNAP were used to further confirm that the formation of adventitious root is mediated by NO. Obviously, SNAP treatment promoted adventitious rooting under osmotic stress. These results implied that NO released from SNP or SNAP might be responsible for the enhancement of adventitious rooting under osmotic stress. In addition, cPTIO significantly reversed the promotive effects of NO on adventitious rooting (**Figure 5** and Supplementary Figure S4).

### Fluorescence Intensity of Ca^2+^ in Hypocotyls during Adventitious Rooting under Osmotic Stress as Affected by SNP

**FIGURE 6:**
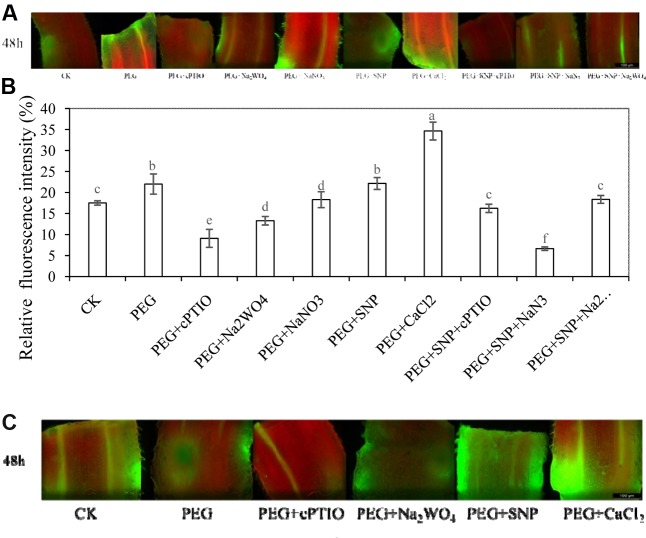
Fluorescence intensity of Ca^2+^ or NO in hypocotyls of cucumber during adventitious rooting under osmotic stress. The primary roots were removed of 6-day-old seedlings. Intracellular Ca^2+^ fluorescence intensity in hypocotyls were treated with distilled water (control), 0.05% (w/v) PEG 6000, PEG + 200 μM cPTIO, PEG + 100 μM Na_2_WO_4_, PEG + 100 μM NaNO_3_, PEG + 10 μM SNP, PEG + 200 μM CaCl_2_, PEG + 10 μM SNP + 200 μM cPTIO, PEG + 10 μM SNP + 10 μM NaN_3_ or PEG + 10 μM SNP + 100 μM Na_2_WO_4_. NO fluorescence intensity in hypocotyls were treated with distilled water (control), 0.05% (w/v) PEG 6000, PEG + 200 μM cPTIO, PEG + 100 μM Na_2_WO_4_, PEG + 10 μM SNP, PEG + 200 μMCaCl_2_. Fluo-3/AM was utilized as the Ca^2+^-sensitive fluorescent probe. DAF-FM DA was utilized to detect endogenous NO of a longitudinal section from the tip of the hypocotyls. Changes in fluorescence intensity of Ca^2+^ **(A)** was monitored by fluorescence microscope after 48 h and expressed as relative fluorescence intensity. The relative fluorescence intensity of intracellular Ca^2+^ **(B)** was analyzed by Image Pro software. Fluorescence intensity expressed as a percentage of control **(B)**. Changes in fluorescence intensity of NO after 48 h of treatment in hypocotyls of cucumber during adventitious rooting under osmotic stress **(C)**.

To investigate whether there is a link between NO and Ca^2+^ during adventitious rooting under osmotic stress, changes in fluorescence intensity of Ca^2+^ in hypocotyls were analyzed (**Figures 6A,B** and Supplementary Figures S5, S7). At 48 h, treatment with PEG + CaCl_2_ in Ca^2+^ fluorescence intensity was significantly higher than that of PEG treatment alone. However, cPTIO, NaN_3_ or Na_2_WO_4_ treatments which may inhibit NO generation significantly impaired the fluorescence intensity of intracellular Ca^2+^ in cucumber hypocotyl at 48 h. Besides, there was no significant difference between PEG treatment and PEG + NaNO_3_ treatment. This gives an indication that NO plays a crucial role in increasing intracellular Ca^2+^ presence. Removing endogenous NO decreased the level of intracellular Ca^2+^which implied NO was responsible for the changes in intracellular Ca^2+^ during adventitious rooting under osmotic stress.

### Changes in Fluorescence Intensity of NO in Hypocotyls of Cucumber during Adventitious Rooting under Osmotic Stress

In order to investigated the connection between NO and Ca^2+^during adventitious rooting under osmotic stress, the level of NO fluorescent intensity was studied (Supplementary Figure S6). As shown in **Figure 6C**, at 48 h, PEG + SNP treatment enhanced the fluorescent intensity of NO production, compared to the control or PEG treatment. Meanwhile, the application of CaCl_2_ relatively increased the accumulation of endogenous NO in cucumber hypocotyl during the development of adventitious root under osmotic stress. The NO scavenger or inhibitor remarkably resulted in a reduction in fluorescence intensity of NO in cucumber. These results implied that there could be a relationship between the amount of endogenous NO and the process of adventitious rooting under osmotic stress.

### Effects of NO and CaCl_2_ on Leaf Water of Cucumber under Osmotic Stress

**FIGURE 7:**
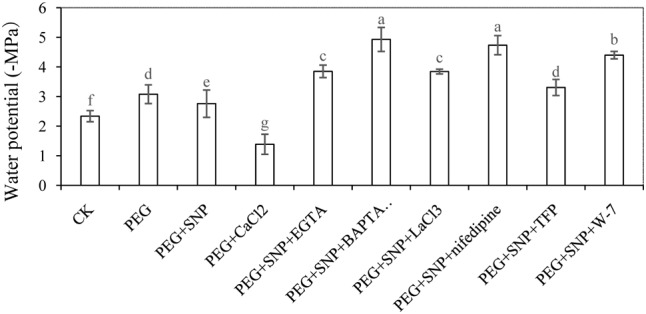
Effects of NO and CaCl_2_ on leaf water of cucumber under osmotic stress. Explants were incubated with distilled water (control) or 0.05% (w/v) PEG 6000, PEG + 10 μM SNP, PEG + 200 μM CaCl_2_, PEG + 10 μM SNP + 100 μM EGTA, PEG + 10 μM SNP + 30 μM BAPTA/AM, PEG + 10 μM SNP + 500 μM LaCl_3_, PEG + 10 μM SNP + 150 μM nifedipine, PEG + 10 μM SNP + 100 μM TFP or PEG + 10 μM SNP + 80 μM W-7. Water potential of leaf in cucumber were determined after 48 h of treatment, respectively. Bars with different lower case letters were significantly different by Duncan’s multiple range test (*p* < 0.05).

**Figure 7** showed that the value of leaf Ψw in PEG treatment is significantly lower than that of the control during adventitious rooting at 48 h. In addition, PEG + SNP or PEG + CaCl_2_ treatment significantly increased leaf Ψw in comparison to PEG-treated leaves only. At 48 h, leaf Ψw of explants treated with PEG increased by 31.8% compared with the control. Application of SNP or CaCl_2_ remarkably increased the Ψw by 10.4%, 55.0% in cucumber leaf, respectively, compared to that of PEG treatment alone. However, EGTA, BAPTA/AM, TFP, W-7, LaCl_3_ or nifedipine treatments resulted in a significant reduction in leaf Ψw during adventitious rooting under osmotic stress. This indicates that Ca^2+^ might be responsible for NO-mediated stress response during adventitious rooting in cucumber under osmotic stress.

### Effects of NO and CaCl_2_ on TBARS, H_2_O_2_ and O_2_^-^ of Cucumber Leaf under Osmotic Stress

**FIGURE 8:**
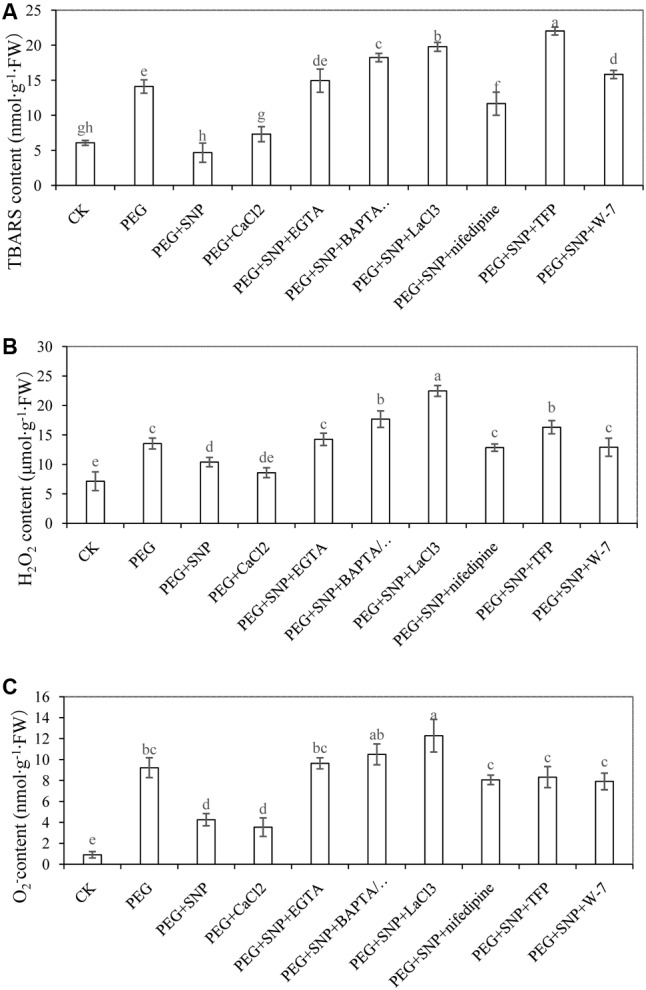
Effects of NO and CaCl_2_ on TBARS **(A)**, H_2_O_2_ **(B)** and O_2_^-^**(C)** of cucumber leaf under osmotic stress. Explants were incubated with distilled water (control) or 0.05% (w/v) PEG 6000, PEG + 10 μM SNP, PEG + 200 μM CaCl_2_, PEG + 10 μM SNP + 100 μM EGTA, PEG + 10 μM SNP + 30 μM BAPTA/AM, PEG + 10 μM SNP + 500 μM LaCl_3_, PEG + 10 μM SNP + 150 μM nifedipine, PEG + 10 μM SNP + 100 μM TFP or PEG + 10 μM SNP + 80 μM W-7. The content of TBARS, H_2_O_2_ and O_2_^-^ in cucumber were determined after 48 h of treatment, respectively. Bars with different lower case letters were significantly different by Duncan’s multiple range test (*p* < 0.05).

As shown in **Figure 8**, PEG treatment significantly increased the concentration of TBARS, H_2_O_2_ and O_2_^-^. The level of reactive oxygen species (ROS) in Ca^2+^ chelators, Ca^2+^ channel inhibitors or CaM antagonist were significantly higher than those of PEG + SNP and PEG + CaCl_2_ treatments. At 48 h, TBARS concentration with PEG treatment was 135.7% higher that of control (**Figure 8A**). However, the concentration of TBARS in PEG + SNP and PEG + CaCl_2_ treatments was significantly reduced by 66.9 and 48.2% compared to PEG treatment.

**Figure 8B** showed that H_2_O_2_ concentration with PEG + SNP and PEG + CaCl_2_ treatments reduced by 23.3 and 36.6% at 48 h, respectively, compared to PEG-treated explants. Conversely, the level of H_2_O_2_ in EGTA, BAPTA/AM, TFP, W-7, LaCl_3_ or nifedipine treatments was expressed at a higher level in comparison with PEG + SNP and PEG + CaCl_2_ treatments at 48 h. The level of O_2_^-^ treated with PEG was significantly enhanced with respect to the control (**Figure 8C**). In addition, a reduction of O_2_^-^ content was observed under PEG + SNP and PEG + CaCl_2_ treatments at 48 h, compared to that of PEG treatment. However, the accumulation of O_2_^-^ content in Ca^2+^ chelators, Ca^2+^ channel inhibitors or CaM antagonist treatment remarkably increased at 48 h.

### Effect of SNP and CaCl_2_ on the Activities of Antioxidant Enzymes during Adventitious Rooting under Osmotic Stress

**FIGURE 9:**
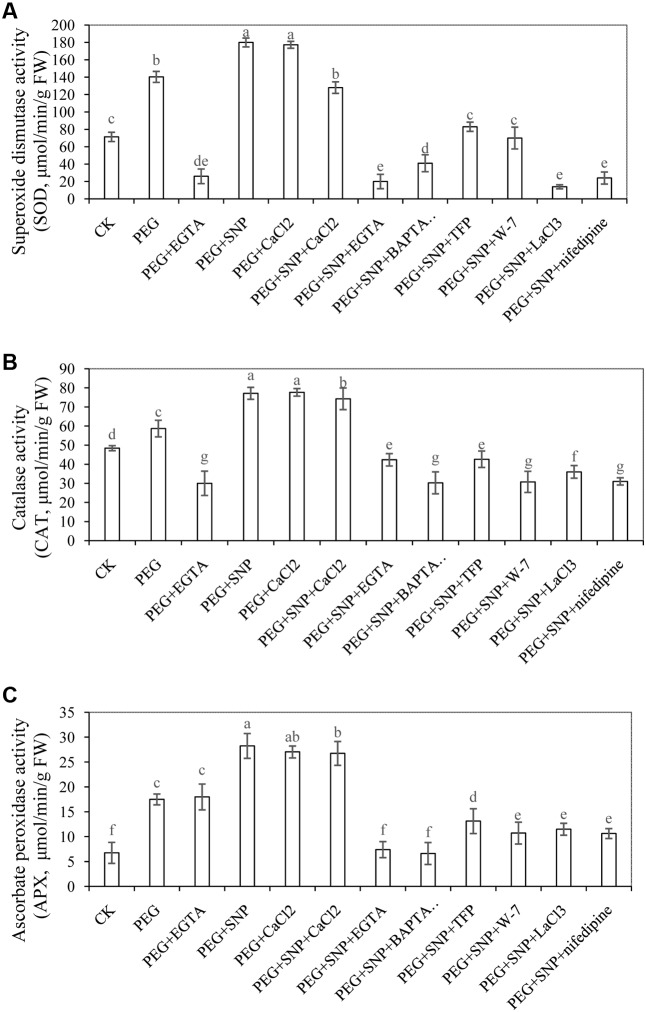
Effects of SNP and CaCl_2_ on the activities of antioxidant enzymes during adventitious rooting under osmotic stress. Cucumber explants were incubated with distilled water (control) or 0.05% (w/v) PEG 6000, PEG + 100 μM EGTA, PEG + 10 μM SNP, PEG + 200 μM CaCl_2_, PEG + 10 μM SNP + 200 μM CaCl_2_, PEG + 10 μM SNP + 100 μM EGTA, PEG + 10 μM SNP + 30 μM BAPTA/AM, PEG + 10 μM SNP + 100 μM TFP, PEG + 10 μM SNP + 80 μM W-7, PEG + 10 μM SNP + 500 μM LaCl3 or PEG + 10 μM SNP + 150 μM nifedipine. Activities of SOD **(A)**, CAT **(B)** and APX **(C)** were determined at 48 h of treatment, respectively. Bars with different lower case letters were significantly different by Duncan’s multiple range test (*p* < 0.05).

To further assess whether alleviation of osmotic stress is related to NO- or CaCl_2_-induced antioxidant defense, we measured the activity of antioxidant enzymes in cucumber explants during adventitious rooting under osmotic stress. The SOD activity in PEG + SNP or PEG + CaCl_2_ was increased by 28.3 and 26.4%, respectively, compared to PEG treatment at 48 h (**Figure 9A**). Meanwhile, the CAT activity in PEG + SNP or PEG + CaCl_2_ treatment at 48 h was 31.5 and 32.3% higher than that in osmotic stress alone (**Figure 9B**). Meanwhile, SNP, CaCl_2_ or SNP + CaCl_2_ treatments resulted in a significant increase of APX activity under osmotic stress (**Figure 9C**). However, during the development of adventitious root, EGTA, BAPTA/AM, TFP, W-7, LaCl_3_ or nifedipine treatments significantly suppressed these antioxidant enzyme activities to levels lower than that of PEG treatment.

### Effects of SNP and CaCl_2_ on Photosynthetic System during Adventitious Rooting under Osmotic Stress

#### Chlorophyll Content

**FIGURE 10:**
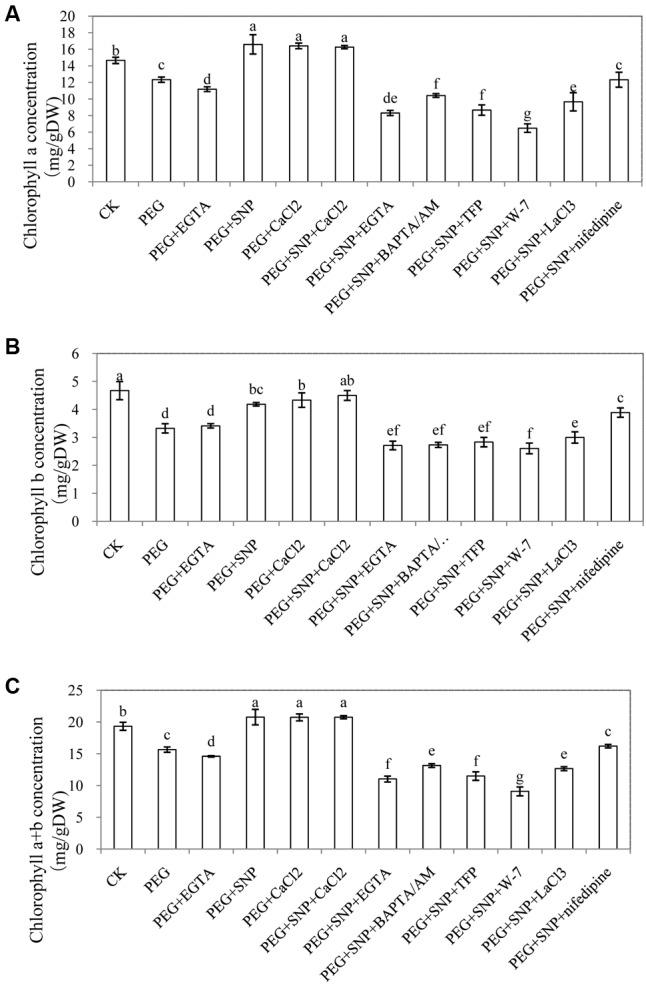
Effects of NO and CaCl_2_ on chlorophyll content of cucumber leaf under osmotic stress. Explants were incubated with distilled water (control) or 0.05% (w/v) PEG 6000, PEG + 100 μM EGTA, PEG + 10 μM SNP, PEG + 200 μM CaCl_2_, PEG + 10 μM SNP + 200 μM CaCl_2_, PEG + 10 μM SNP + 100 μM EGTA, PEG + 10 μM SNP + 30 μM BAPTA/AM, PEG + 10 μM SNP + 100 μM TFP, PEG + 10 μM SNP + 80 μM W-7, PEG + 10 μM SNP + 500 μM LaCl_3_ or PEG + 10 μM SNP + 150 μM nifedipine. Leaf chlorophyll a **(A)**, chlorophyll b **(B)** and chlorophyll a+b **(C)** content was measured after 48 h of treatment. The values (mean ± SE) are the average of three independent experiments. Bars with different lower case letters were significantly different by Duncan’s multiple range test (*p* < 0.05).

Osmotic stress decreased chlorophyll content in our experiment (**Figure 10**). The chl a, chl b, chl (a + b) concentration in cucumber leaves from explants that were treated with in SNP, Ca^2+^ or SNP + CaCl_2_ treatments significantly increased, compared with those from PEG and PEG + EGTA treatment. These results suggested that application of NO or CaCl_2_ was more effective in retarding the loss of chlorophyll and in improving photochemistry under osmotic stress. However, EGTA, BAPTA/AM, TFP, W-7, LaCl_3_ and nifedipine treatments decreased the level of chl a, chl b and chl (a + b), compared to explant exposed to SNP, CaCl_2_ or SNP + CaCl_2_ treatments. Meanwhile, the concentrations of chl a, chl b, chl (a + b) in explants treated with EGTA, BAPTA/AM, TFP, W-7, LaCl_3_ and nifedipine were significantly lower than those of PEG treatment which indicated that Ca^2+^/CaM might be involved in NO-regulating the increases in chlorophyll content.

#### Effect of SNP or CaCl_2_ on Photochemistry under Osmotic Stress

Compared to the control, PEG and PEG + EGTA treatment decreased the value of Fv/Fm (**Table 1**). However, SNP treatment or CaCl_2_ treatment increased Fv/Fm by 9.2 and 8.2% higher than that of control, respectively. Moreover, EGTA, BAPTA/AM, TFP or W-7 treatments resulted in 12.6, 15.5, 14.1, and 14.6% reduction in Fv/Fm, respectively, compared to osmotic stress. LaCL_3_ and nifedipine treatment significantly reduced Fv/Fm by 46.4 and 37.9%, respectively, compared to that of PEG + SNP treatment. In addition, the effect of different treatments on ΦPSII and qP were similar to that of Fv/Fm (**Table 1**). PEG treatment significantly increased NPQ compared with control. However, PEG + SNP or PEG + CaCl_2_ treatments significantly decreased NPQ. There was a significant increase in NPQ level after removing the endogenous Ca^2+^/CaM. These results implied that NO or Ca^2+^ had a positive role in improving the development of adventitious root by regulating chlorophyll florescent parameters.

**Table 1:** Effects of SNP or CaCl_2_ on photochemistry under osmotic stress.

Treatments	Fv/Fm	ΦPSII	qP	NPQ
CK	0.783d	0.597c	0.647i	0.467l
PEG	0.683e	0.533d	0.583e	0.713f
PEG + EGTA	0.630f	0.452g	0.498j	0.668h
PEG + SNP	0.855a	0.667a	0.723a	0.527i
PEG + CaCl_2_	0.847b	0.653b	0.717b	0.520j
PEG + SNP + CaCl_2_	0.827c	0.653b	0.703c	0.517k
PEG + SNP + EGTA	0.597g	0.463e	0.503d	0.757b
PEG + SNP + BAPTA/AM	0.577k	0.447h	0.513f	0.767a
PEG + SNP + TFP	0.587i	0.453f	0.507h	0.747e
PEG + SNP + W-7	0.583j	0.437i	0.508g	0.750d
PEG + SNP + LaCl_3_	0.558l	0.350k	0.396k	0.671g
PEG + SNP + nifedipine	0.591h	0.413j	0.304l	0.755c


*Explants were incubated with distilled water (control) or 0.05% (w/v) PEG 6000, PEG + 100 μM EGTA, PEG + 10 μM SNP, PEG + 200 μM CaCl_2_, PEG + 10 μM SNP + 200 μM CaCl_2_, PEG + 10 μM SNP + 100 μM EGTA, PEG + 10 μM SNP + 30 μM BAPTA/AM, PEG + 10 μM SNP + 100 μM TFP, PEG + 10 μM SNP + 80 μM W-7, PEG + 10 μM SNP + 500 μM LaCl_3_ or PEG + 10 μM SNP + 150 μM nifedipine. Parameters of chlorophyll fluorescence with different treatments were conducted after 48 h of treatment. Values followed by a different lower-case letter in the same column were significantly different by Duncan’s multiple range test at *p* < 0.05.*

## Discussion

Previous studies have indicated that NO and Ca^2+^, as signaling molecules, might be involved in plants response to abiotic stress (Shan et al., 2015; Zou et al., 2015; Silveira et al., 2017a). However, whether there is a relationship between NO and Ca^2+^/CaM in the development of adventitious root under osmotic stress has not received the needed research attention. In this study, we have found that Ca^2+^/CaM interactions were involved in NO-induced adventitious rooting process in cucumber under osmotic stress.

### Actions and Relationships between NO and Ca^2+^on Promoting Adventitious Rooting under Osmotic Stress

The number and length of adventitious roots in cucumber was retarded by different concentrations of PEG treatment. However, when exogenous SNP or CaCl_2_ were applied under osmotic stress, root number and length was significantly increased to levels higher than those of PEG treatment (**Figures 2**, **3**). According to previous report, NO promoted adventitious root formation in marigold under drought stress (Liao et al., 2012a). Ca^2+^ was also found to have induced the adventitious rooting process in marigold explants under normal condition (Liao et al., 2012b). According to our results (**Figures 2**, **3**), SNP or Ca^2+^ had the dose-dependent effect on induction of adventitious rooting in cucumber under osmotic stress. In addition, results from PEG + K_4_Fe(CN)_6_, PEG + SNAP and PEG + SNP + cPTIO treatments further confirmed the role of NO released from SNP and SNAP (**Figure 5**). These results indicated that suitable dosages of NO or Ca^2+^ might alleviate the injury due to osmotic stress on adventitious rooting. Previous researches have suggested that NO or Ca^2+^ played critical roles in enhancing stress resistance through mediating antioxidant defense system, protecting the structure of cell membrane and chlorophyll function as well as maintaining ion homeostasis in plants (Shan et al., 2015; Zou et al., 2015; Li et al., 2016). Recently, the crosstalk between NO and Ca^2+^ under abiotic stress in plants was reported. For example, NO might mediate Ca^2+^-regulated antioxidant enzymes activities to respond against high irradiance -induced oxidative stress (Xu et al., 2016). Similarly, Lang et al. (2014) investigated the functional relationship between NO and Ca^2+^ mediating root ion fluxes under salt stress. In another experiment with explants treated with EGTA, BAPTM/AM, LaCl_3_, nifedipine, TFP and W-7, the formation of NO-induced adventitious root was significantly retarded under osmotic stress (**Figure 4**). These results revealed that inhibition of endogenous Ca^2+^/CaM might completely inhibit the NO-induced adventitious rooting under osmotic condition. Previous study on rice indicated that Ca^2+^ was involved in NO-induced lateral root formation (Chen and Kao, 2012) and Ca^2+^ inhibitors (EGTA and BAPTA/AM) or CaM antagonists (TFP and W-7) were found to have suppressed the positive effects of NO and H_2_O_2_ on adventitious rooting of marigold under stress-free condition (Liao et al., 2012a). Additionally, Lanteri et al. (2006) also found that Ca^2+^ might be involved in NO- or auxin-induced adventitious root formation through activating CDPK activity and the application of Ca^2+^ channel inhibitors significantly suppressed NO- and IAA-induced development of adventitious rooting which indicated that Ca^2+^ is required for the adventitious rooting process. Our results further revealed that Ca^2+^/CaM played an essential role in NO-induced adventitious rooting under abiotic stress. Ca^2+^/CaM might be a downstream signaling in the NO-induced adventitious rooting under osmotic stress.

The application of SNP elevated intracellular Ca^2+^ in cucumber hypocotyls under osmotic stress which indicated that the promotive effect of NO under osmotic stress was dependent on increasing the production of Ca^2+^ in plant cell. Abdul-Awal et al. (2016) reported that NO could mediate increases of [Ca^2+^]cyt through activity of ADP-Ribosyl Cyclase in *Arabidopsis*. Ma et al. (2008) also suggested that NO synthesis might be activated by Ca^2+^ through CaMs in *Arabidopsis*. These studies suggested that NO and Ca^2+^ may modulate each other’s level to regulate the plant developmental and physiological processes. In our study, SNP + cPTIO, SNP + NaN_3_ and SNP + Na_2_WO_4_ significantly inhibited the level of endogenous Ca^2+^ during adventitious root formation (**Figures 6A,B**). In addition, treatment with PEG + cPTIO or PEG + Na_2_WO_4_ significantly decreased the production of endogenous Ca^2+^ in hypocotyl as well. Therefore, Ca^2+^ might be as downstream of NO in the development of adventitious root under osmotic stress. In previous studies, hydrogen gas (H_2_) (Zhu et al., 2016), carbon monoxide (CO) (Chen et al., 2017) and H_2_O_2_ (Xuan et al., 2008; Yang et al., 2013) were also suggested to be involved in plants adventitious rooting. These signaling molecules may mediate each other through activation of receptors or targeted protein to affect the process of adventitious rooting in plants. Additionally, Endogenous NO might accumulate during the development of adventitious root (**Figure 6C**). Previous studies showed that NO could be an essential signaling molecule to regulate the development and growth of adventitious roots in plants (Lanteri et al., 2008; Liao et al., 2012a). Pagnussat et al. (2002) also suggested that NO might be involved in auxin-induced adventitious root formation through transient NO accumulation in cucumber explants. These results indicated that NO is responsible for the adventitious root organogenesis. Silveira et al. (2017a) found that drought-tolerant sugarcane genotype could accumulate more NO than the sensitive one, suggesting that there might exist an association between drought-tolerance and NO production and metabolism. Interestingly, CaCl_2_ treatment also increased the content of endogenous NO as well which implied that there might be a close interplay between NO and Ca^2+^ during the development of adventitious roots in cucumber under stress condition.

### Activation of Antioxidative Response during Adventitious Rooting under Osmotic Stress

An increase in the ROS level might induce membrane lipid peroxidation in cells (Panyuta et al., 2016). In order to investigate the effects of SNP and CaCl_2_ on the development of adventitious root under osmotic stress, the changes in TBARS in cucumber explants were analyzed. At 48 h, PEG treatment significantly enhanced the concentration of TBARS in comparison with the control (**Figure 8A**), which implied that osmotic stress might induce the accumulation of TBARS (Jaleel et al., 2007). In addition, PEG + SNP and PEG + CaCl_2_ treatments significantly reduced the content of TBARS compared to PEG treatment (**Figure 8A**). Previous study showed NO could protect sunflower leaves under Cd-induced oxidative stress by decreasing TBARS content (Laspina et al., 2005). Besides, Larkindale and Knight (2002) also suggested that inhibiting the endogenous Ca^2+^ content could increase the oxidative damage under heat stress by increasing the TBARS content in *Arabidopsis*. Our results showed that the application of SNP or CaCl_2_ significantly decrease TBARS content in cucumber explant under osmotic stress (**Figure 8A**). However, the TBARS content of EGTA, BAPTA/AM, TFP, W-7, LaCl_3_ or nifedipine treatments were significantly higher than those of SNP or CaCl_2_ treatments. These results indicated that removal of endogenous Ca^2+^ during NO-induced adventitious rooting under osmotic stress probably increased the level of membrane lipid peroxidation, hence, aggravated oxidative stress and caused cell death or the decrease of relative antioxidant enzymes activities (Liu et al., 2013; Chen et al., 2015).

H_2_O_2_ or O_2_^-^, which was a form of ROS, also caused oxidative damage in plants under stressful condition (Romero-Puertas et al., 2004; Cho and Seo, 2005). Wang and Yang (2005) found that SNP pre-treatment significantly inhibited the generation of O_2_^⋅-^ and H_2_O_2_ in the roots of *Cassia tora* L under Aluminum (Al) stress. Another research also found that there is a reduction in the content of H_2_O_2_, TBARS and other ROS in CaCl_2_ treatment in soybean seedlings under salt stress (Arshi et al., 2010). Our results showed that SNP or CaCl_2_ treatments under osmotic stress significantly reduced the production of H_2_O_2_ or O_2_^-^ during the process of adventitious root development (**Figures 8B,C**). However, the application of Ca^2+^ chelators, Ca^2+^ channel inhibitors or CaM antagonist might keep a higher level H_2_O_2_ or O_2_^-^ content than those of SNP or CaCl_2_ treatment. These findings also suggest that NO or Ca^2+^ might reverse the damage of osmotic stress during adventitious rooting by blocking the generation of ROS in cucumber explants.

There are several reports that NO or Ca^2+^ might have the capacity to regulate the antioxidant system by increasing the activities of antioxidant enzymes under various stresses (Shi K. et al., 2015; Zou et al., 2015; Silveira et al., 2017b). Our results showed that SNP, CaCl_2_ or SNP + CaCl_2_ treatment significantly increased the activities of antioxidant enzymes during the development of adventitious root under osmotic stress. SOD, CAT and APX activities in cucumbers treated with SNP, CaCl_2_ or SNP + CaCl_2_ were higher than those of PEG treatment (**Figure 9**). Li et al. (2016) suggested that Ca^2+^ could alleviate the inhibition of Cd on the root growth by reducing oxidative injuries which indicated that there could be a relationship between the root growth and oxidative system. Moreover, El-Shabrawi et al. (2010) also found that there was a positive relationship between increased antioxidant defense mechanism and the reduction of oxidative damage. The increased activities of antioxidant enzymes increased oxidation resistance by decreasing ROS generation rate during adventitious rooting process.

### Enhancement of Water Retention and Photosynthetic Activity under Osmotic Stress

In order to confirm the influence of PEG-induced osmotic stress, leaf Ψw in cucumber was determined in our experiment (**Figure 7**). Previous study found that Cd might decrease leaf Ψw and relative water content (RWC) in bean plants (Barcelo et al., 1986). Wilson et al. (2014) also suggested that plastid osmotic stress might significantly decreased water potential in *Arabidopsis*. In our study, greater reduction in leaf Ψw was observed with PEG treatment compared to the control (**Figure 7**). The leaf Ψw was significantly increased by exogenous supplied SNP or CaCl_2_ under osmotic stress. Recent research suggested NO might alleviate water deficit stress through increasing leaf Ψw and RWC in *Cakile maritima* (Jday et al., 2016). Our results indicated that application of exogenous NO might increase the water potential in cucumber under stress condition to alleviate the damage of osmotic stress during adventitious rooting. Meanwhile, inhibition of endogenous Ca^2+^ accumulation might reverse the positive effect of NO on alleviating the osmotic damage during the development of adventitious root in cucumber.

Górnik et al. (2008) found that drought stress reduced chlorophyll content in the leaves during rooting of grapevine cuttings. In addition, Humphries and Thorne (1964) suggested that photosynthesis was retarded when root growth was restricted by application of kinetin. These results imply that there is a link between photosynthesis and rooting. Uchida et al. (2002) found that suitable concentration of NO restored the chlorophyll levels in rice leaves and protected photosystem II (PSII) to maintain a relatively high activity under salt and heat stresses. Also, Silveira et al. (2016) indicated that exogenous NO might improve photosynthesis under water deficit and Tiwari et al. (2016) noticed that Ca^2+^ application alleviated the reduction of chl a under heat stress. The application of CaCl_2_ enhanced photosynthesis by increasing the contents of chl a, chl b, and chl (a + b) in *Zoysia japonica* under drought stress (Xu et al., 2013). In our study, the content of chl a, chl b, or chl (a + b) in cucumbers treated with PEG was significantly lower than those of control, while application of SNP or CaCl_2_ under osmotic stress increased the concentrations of chl a, chl b, or chl (a + b) to levels higher than PEG treatment alone (**Figure 10**). These findings indicated that NO or CaCl_2_ treatment might significantly prevent the degradation of chlorophyll in cucumber in order to promote photosynthesis during adventitious rooting under osmotic stress. The EGTA, BAPTA/AM, TFP, W-7_,_ LaCl_3_ and nifedipine_,_ however, caused significant degradation of chlorophyll content in the leaves. It has been reported that abiotic stress led to decreases in photosynthetic rates by affecting chlorophyll fluorescence parameters in plants (Hou et al., 2016). In our study, PEG treatment significantly decreased the value of Fv/Fm, ΦPSII or qP but increased NPQ. However, SNP or CaCl_2_ treatment increased Fv/Fm, ΦPSII or qP which implied that NO or CaCl_2_ could reduce damage to photosynthesis under osmotic stress, maintaining the photochemical activity. Zhang et al. (2012) found that NO alleviated iron-deficiency in peanut leaves via increasing Fv/Fm and ΦPSII which suggested that NO had a protective effect on photosystem II (PSII) in plants. In addition, Zhao and Tan (2005) reported that exogenous Ca^2+^ also played a crucial role in protecting photochemistry in wheat plants under heat and high irradiance stresses. However, EGTA or LaCl_3_ treatment had the opposite effect which indicated that endogenous Ca^2+^ might be involved in mediating photosynthesis in wheat. These results indicated that there might be a positive relationship between adventitious rooting and photosynthetic level. Besides, Ca^2+^ might be a downstream molecule involved in NO-induced formation of photosynthetic pigments and increases of photochemical activity in order to promote adventitious rooting under osmotic stress.

## Conclusion

The results of our experiments have shown that exogenous application of NO and CaCl_2_ alleviated osmotic stress and promoted the development of adventitious roots in cucumber under stressful conditions. Our data also revealed that Ca^2+^/CaM might be downstream molecules of NO signaling pathway, protecting photosynthetic system and stimulating the antioxidant defense system. As there is a complex interaction between NO and Ca^2+^/CaM in the adventitious rooting process under abiotic stress, further work should focus on the molecular mechanism of the crosstalk between NO and Ca^2+^/CaM during signaling transduction under various stresses.

## Author Contributions

WL designed the experiments; LN and JY performed the experiments; WL, LN, and MZ performed data analysis; WL, LN, and JY wrote the manuscript; WL, JhY, and MD edited the manuscript.

## Conflict of Interest Statement

The authors declare that the research was conducted in the absence of any commercial or financial relationships that could be construed as a potential conflict of interest.
