# Supplementary material for: Calcium and Calmodulin Are Involved in Nitric Oxide-Induced Adventitious Rooting of Cucumber under Simulated Osmotic Stress
Source: Front Plant Sci. 2017 Sep 27;8:1684. doi: 10.3389/fpls.2017.01684 (PMC5623940; doi:10.3389/fpls.2017.01684)
Supplement: Supplementary file 1 [file Image_1.PDF]

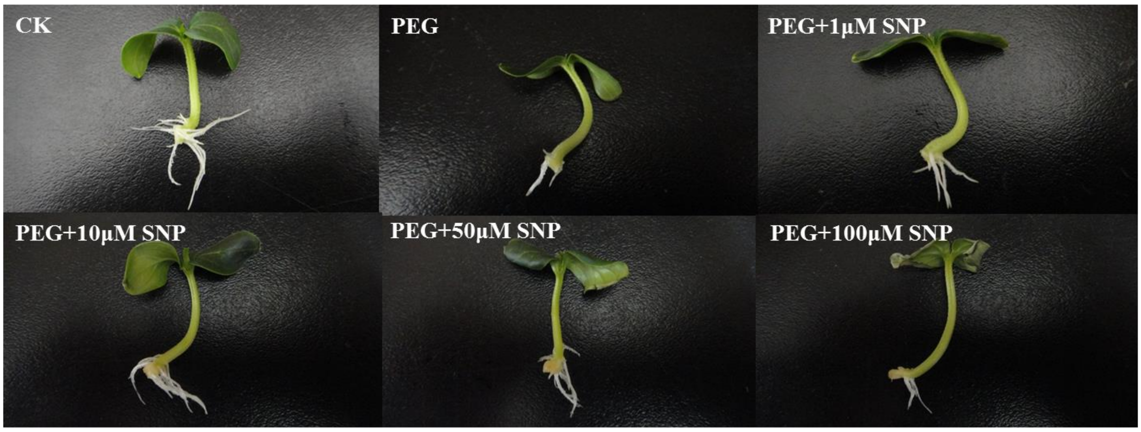

IMAGE1 | Effect of different concentrations of SNP on adventitious root development in cucumber explants under osmotic stress. Explants were incubated for 6 d with distilled water (control) or 0.05% (w/v) PEG 6000 plus different concentrations of SNP. Photographs were taken after 6 days of treatments.
