# Supplementary material for: Calcium and Calmodulin Are Involved in Nitric Oxide-Induced Adventitious Rooting of Cucumber under Simulated Osmotic Stress
Source: Front Plant Sci. 2017 Sep 27;8:1684. doi: 10.3389/fpls.2017.01684 (PMC5623940; doi:10.3389/fpls.2017.01684)
Supplement: Supplementary file 3 [file Image_3.PDF]

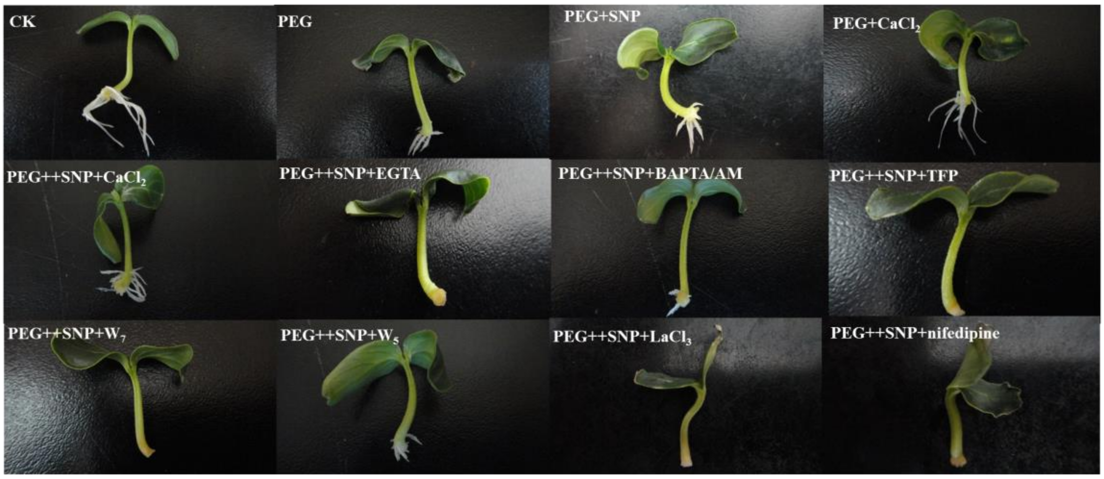

IMAGE 3 | Effect of EGTA, BAPTA/AM, TFP, W-7, W-5, $LaCl_3$ or nifedipine on adventitious rooting under osmotic stress. Explants were incubated for 6 d with distilled water (control) or 0.05% (w/v) PEG 6000PEG+10 μM SNP, PEG+200 μM$CaCl_2$, PEG+10 μM SNP +200 μM $CaCl_2$, PEG+10 μM SNP+100 μM EGTA, PEG+10 μM SNP+30 μM BAPTA/AM, PEG+10 μM SNP+100 μM TFP, PEG+10 μM SNP+80 μM W-7, PEG+10 μM SNP+80 μM W-5, PEG+10 μM SNP+500 μM $LaCl_3$ or PEG+10 μM SNP+150 μM nifedipine. Photographs show hypocotyls explants after 6 d of the treatments indicated.
