# Supplementary material for: Calcium and Calmodulin Are Involved in Nitric Oxide-Induced Adventitious Rooting of Cucumber under Simulated Osmotic Stress
Source: Front Plant Sci. 2017 Sep 27;8:1684. doi: 10.3389/fpls.2017.01684 (PMC5623940; doi:10.3389/fpls.2017.01684)
Supplement: Supplementary file 4 [file Image_4.PDF]

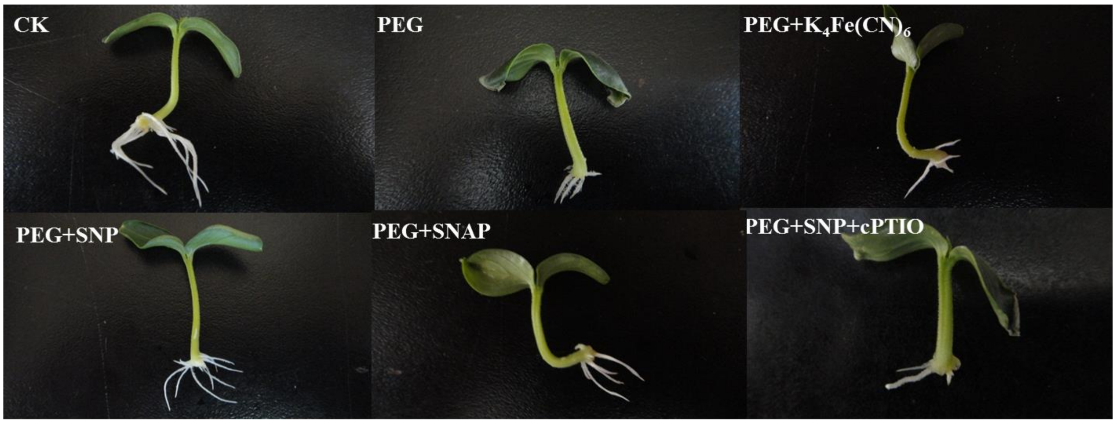

IMAGE 4 | Effect of $K_4Fe(CN)_6$, SNAP or cPTIO on adventitious rooting under osmotic stress. Explants were incubated for 6 d with distilled water (control) or 0.05% (w/v) PEG 6000, PEG+50 μM $K_4Fe(CN)_6$, PEG+10 μM SNP, PEG+50 μM SANP, PEG+10 μM SNP+200 μM cPTIO. Photographs show hypocotyls explants after 6 d of the treatments indicated.
