# Supplementary material for: Calcium and Calmodulin Are Involved in Nitric Oxide-Induced Adventitious Rooting of Cucumber under Simulated Osmotic Stress
Source: Front Plant Sci. 2017 Sep 27;8:1684. doi: 10.3389/fpls.2017.01684 (PMC5623940; doi:10.3389/fpls.2017.01684)
Supplement: Supplementary file 7 [file Image_7.PDF]

Supplementary Material

Calcium and calmodulin are involved in nitric oxide-induced adventitious rooting of cucumber under simulated osmotic stress

Lijuan Niu, Jian Yu*, Jihua Yu, Meiling Zhang, Mohammed Mujitaba Dawuda*

*** Correspondence:** Dr. *Weibiao Liao*

Corresponding Author: liaowb@gsau.edu.cn

Supplementary Figures

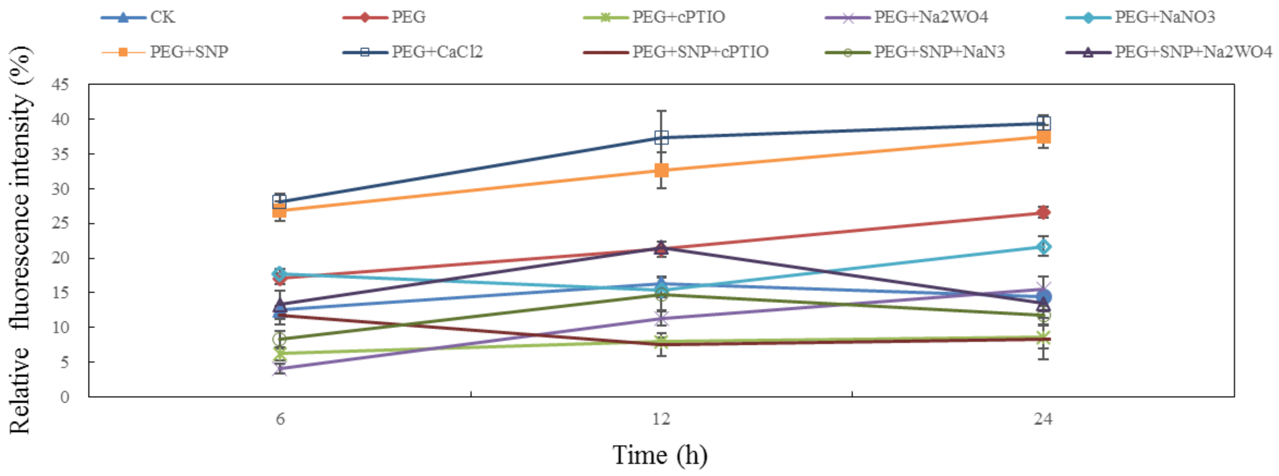

Supplementary Figure 1. Fluorescence intensity of Ca^{2+} in hypocotyls during adventitious rooting under osmotic stress as affected by SNP. The primary roots were removed of 6-day-old seedlings. Intracellular Ca^{2+} fluorescence intensity in hypocotyls were treated with distilled water (control), 0.05% (w/v) PEG 6000, PEG+200 μM cPTIO, PEG+100 μM Na_2WO_4, PEG+100 μM $NaNO_3$, PEG+10 μM SNP, PEG+200 μM $CaCl_2$, PEG+10 μM SNP+200 μM cPTIO, PEG+10 μM SNP+10 μM NaN_3 or PEG+10 μM SNP+100 μM Na_2WO_4.
